# Intravenous cannulation and administration of contrast media by radiographers: a literature review to guide the training and practice in Zambia

**DOI:** 10.4314/ahs.v22i2.72

**Published:** 2022-06

**Authors:** Osward Bwanga, Raphael Musoko Kayembe, James Maimbo Sichone

**Affiliations:** 1 Midland Regional Hospital at Tullamore, Radiology Department, Co. Offaly, Ireland; 2 Blackrock Clinic, Radiology Department, Dublin, Ireland; 3 University of Zambia, School of Health Sciences, Radiography Section, Lusaka, Zambia

**Keywords:** Contrast media, intravenous cannulation, radiographer, radiologist, Zambia

## Abstract

**Background:**

There has been a demand for radiographers in Zambia to perform intravenous (IV) cannulation and administration of contrast media, a role which, traditionally, was radiologists’. This demand is due to a shortage of radiologists and an increase in demand for imaging examinations. This review aimed at synthesising relevant literature related to IV cannulation and administration of contrast media by radiographers to guide the training and practice in Zambia.

**Methods:**

A structured literature search was conducted in three online databases (PubMed/MEDLINE, CINAHL, and ScienceDirect), radiography journals, and cited references to identify research studies on IV cannulation and administration of contrast media by radiographers.

**Results:**

Seven studies were identified and included in this review. The findings are summarised under six themes: benefits and challenges, adoption of the extended role, infection control, safety and complications, medico-legal issues, and education and training. Our findings revealed that radiographer-led IV cannulation and administration of contrast media contribute positively to the management of imaging patients.

**Conclusion:**

The themes identified in this review could provide a template of where to base the establishment of the training programme and local guidelines. Before extending the role of radiographers, the scope of practice should be extended, and accredited training programme and local guidelines should be put in place.

## Introduction

The practice of radiography is developing and changing in Zambia and globally. Radiographers are taking on roles that were traditionally performed by radiologists and other health professionals. These responsibilities and skills that extend beyond the statutory responsibilities and competencies at the point of professional registration have been termed as role extension^1^. Role extension in radiography started in the United Kingdom (UK) due to a shortage of radiologists and an increase in demand for imaging examinations 1,2,3. Most developed countries have extended the individual scope of practice of radiographers to include intravenous (IV) cannulation and administration of contrast media to patients undergoing medical imaging examinations 4–7. In African countries, role extension for radiographers is in the early stages of development.

In the radiology department, contrast media are used daily to aid in the visualisation of most body organs and blood vessels. The contrast media used include iodinated contrast agents for X-ray and computed tomography (CT) based studies, gadolinium-based contrast agents for magnetic resource imaging (MRI), and microbubbles for ultrasonography 8. The development and increase in the use of imaging modalities, such as CT and MRI, for diagnosis of diseases have resulted in increased use of contrast media. In England, approximately 5.5 million CT and 3.7 million MRI imaging examinations were performed from March 2018 to March 2019 9. Similarly, in the USA half of the approximately 76 million CT and 34 million MRI examinations performed each year include the administration of IV contrast media 10. The increase in demand is not matching with the number of radiologists.

There is a growing body of evidence that radiographer-led IV cannulation and contrast media administration contribute positively to patient management as radiographers are directly involved with imaging examinations. The benefits reported in the literature include improvements in departmental workflow, reduction of patients' waiting time, and relieving pressure from radiologists who can then concentrate on complex tasks, such as interventional procedures^11^–^13^. The Zambian healthcare system can benefit from formally allowing radiographers to perform IV cannulation and administer contrast media to patients for imaging examinations such as intravenous urogram (IVU), CT and MRI. This is due to the increase in demand for imaging examinations, coupled with the critical shortage of radiologists^14^. Therefore, this review is aimed at synthesising literature related to IV cannulation and administration of contrast media by radiographers to guide the training and practice in Zambia.

## Methods

A structured literature search was conducted in October and November 2020, using a combination of the keywords: “radiographers”, “intravenous (IV) cannulation” and “contrast media”. This involved a three-step search strategy: searching databases, hand searching, and citation chaining. In the first step, the search was conducted in three databases: CINAHL, PubMed/MEDLINE, and ScienceDirect. The second step involved manual searching of electronic tables with radiography content and the internet to supplement databases. The journals searched include the South African Radiographer, Radiography (UK), and Journal of Medical Radiation Sciences. In the final stage, bibliographies of identified main studies were looked at to identify related articles not found during database and hand searching.

The inclusion criteria included peer-reviewed primary research studies on IV cannulation and administration of contrast media by radiographers. Due to limited literature on this topic, all available studies (qualitative, quantitative, and mixed methods) were considered in this review. There was no time frame because role extension amongst radiographers is a new practice. However, studies not published in English were excluded due to a lack of resources for translation. Reviews, case reports, and opinion articles were also excluded as per the objective of this review which focuses on primary research studies.

## Results

**Figure 1:**
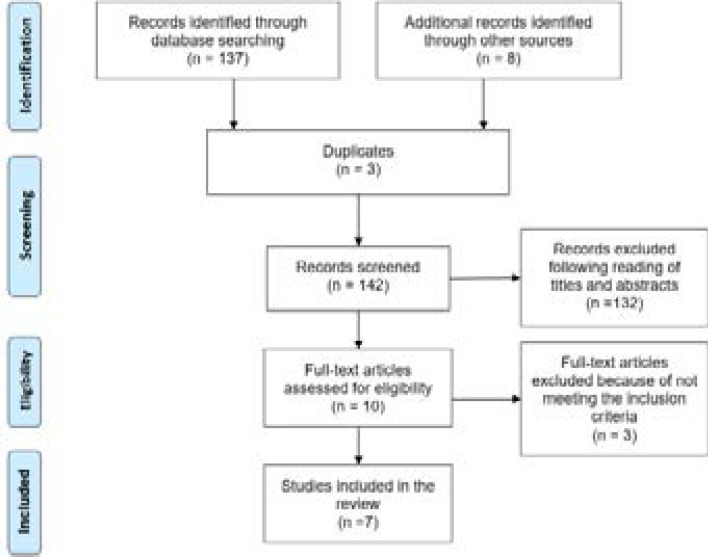
PRISMA flowchart showing the selection of relevant research studies

A total of 145 articles were retrieved. Three were removed due to duplication and 134 articles were excluded following a reading of titles and abstracts. The remaining ten articles were retrieved for eligibility assessment. Three articles were excluded because one was a discussion paper 4, and the other two 15,16 did not relate to radiographers performing IV cannulations and administration of contrast agents. Seven studies 11,17–22 remained and were included in this review. The results of the search and selection of studies are presented in Figure 1.

Amongst the seven studies eligible for this review, six were surveys 11,17, 19–22 and an observational study 18. They were conducted in the Republic of Ireland (N=2), Australia (N=1), South Africa (N=3), and Malaysia (N=1). The rest of the characteristics are presented in Table 1.

**Table 1:** Characteristics of included primary research studies (N=7)

No	Author	Year	Title	No. of participants	Main findings	Country
1	Toh et al., 17	2007	An investigation into the perceptions of radiographers in the Greater Sydney metropolitan area on performing intravenous contrast media administration	90	Most radiographers were in favour of performing contrast media injections. Benefits identified include aid in department workflow, and free up radiologists. Challenges identified include increased workload and medico-legal implications. The need for appropriate rewards was suggested.	Australia
2	Suing & Davis 18	2009	Would you like an infection with your injection? A study investigating infection control and administering policies regarding radiographers performed intravenous injections	9	Radiographers did not adhere to hospital infection control guidelines when inserting IV cannulas.	Ireland
3	Munro et al., 19	2012	An analysis of the need for accredited training on the administration of intravenous contrast media by radiographers: results of an online survey	177	Training should include the administration of contrast media and the resuscitation of a patient. The main benefit identified was improved service delivery. The main disadvantage was the risk of litigation. Because of this, respondents suggested the need for malpractice insurance.	South Africa
4	Zain et al., 11	2016	Perception of radiographers on performing intravenous contrast media administration	110	Radiographers were willing to perform IV injection if appropriate training is given. The main benefits identified were improvement in departmental flow, reducing patients' waiting time, and freeing up radiologists.	Malaysia
5	Cleary et al., 20	2017	An investigation into current protocols and radiographer opinions on contrast extravasation in Irish departments	24	CT departments had written protocols in place. Radiographers indicated that contrast extravasation was more likely in patients with fragile veins and during CT angiography examinations. The workload was identified as a factor impacting patient care.	Ireland
6	Koch et al., 21	2017	Training requirements for the administration of intravenous contrast media by radiographers: Radiologists' perspective	59	The training should include anatomy and physiology of the vascular system, pharmacology of contrast media, practical training on resuscitation and basic life support and rights of patients as well as the responsibilities of a healthcare professional. Assessment should include both theory and practicals.	South Africa
7	Koch et al.,^22^	2018	Medicolegal responsibilities for the administration of intravenous contrast media by radiographers: Radiologists' perspectives.	47	Radiographers should be responsible for obtaining informed consent and deciding on the cannulation site. Radiologists should remain responsible for decisions regarding the type and dose of IV contrast media and managing adverse reaction.	South Africa

The findings of this review are summarised under six themes: benefits and challenges, adoption of the extended role, infection control, patient safety and complications, medico-legal issues, and education and training. These themes forms sub-sections of the discussion.

## Discussion

### Benefits and challenges of radiographers led IV cannulation and administration of contrast media

Three studies 11,17,19 identified the main benefits for radiographer-led IV cannulation and administration of contrast media. The benefits could be grouped under four categories: to the patients, radiographers, radiologists, and radiology departments. To patients, there is a reduction in the waiting time and improved patient care^19^. To radiographers, there is an improvement in the status of radiographers, and job satisfaction 19. To radiologists, there is a reduction of workload 11,17. Lastly, to the radiology department, there is an improvement in the workflow and patient throughput, and increased imaging service delivery 11,17. These findings were also identified in a literature review conducted by Thom 3 on the benefits of advanced practice in radiography to healthcare delivery. Advanced practice is another term for role extension, where radiographers take on roles previously undertaken by radiologists, such as IV cannulation.

Three studies 17,19,20 also reported the challenges of radiographers taking up the role of IV cannulation and administration of contrast media. The challenges identified include additional workload and increased work-related stress 17,19. It is, therefore, important to plan and provide appropriate remuneration to radiographers to equate to the additional responsibilities. This also requires an increase in the number of radiographers to fill up the gap created. The other challenge reported in the literature is a feeling amongst radiologists of being threatened by losing one of their responsibilities 2, 13,23. It should be mentioned that radiologists have a key role to play in supporting radiographers undertaking advanced practice by providing training and by acting as mentors. Therefore, radiologists should be fully involved during the planning and implementation stages for the success of the extension role practice.

The Zambian context is similar to those presented in the literature. The benefits outlined in the literature that would accrual by the implementation of this extended role are also true for Zambia. Radiology services in Zambia, especially in the major hospitals, are characterised by long queues and delayed imaging service delivery. Contributing to this situation are delays and the non-availability of skilled manpower and imaging materils.

### Adoption of IV cannulation and administration of contrast media

The role of radiographers performing IV cannulation and administration of contrast media is well established in Australia 17 and Ireland 20. Despite the non-identification of any UK-based study in our review, this role is also well established in the UK 3,4. This review also found that some radiographers in South Africa are performing this new role outside the scope of their practice 19. Globally, radiographers have professional duties and responsibilities in terms of conduct, performance, and ethics, including a requirement to undertake only tasks they are competent and licenced to undertake 24–27. This means that radiographers must work within the scope of practice. Therefore, extending the scope of practice through regulators, such as the Health Professions Council of Zambia (HPCZ), is the first step in advanced practice.

It is best practice to conduct periodical clinical audits following the implementation of radiographer-led IV cannulation and administration of contrast media to ensure that radiographers are adhering to the protocol 7,28. The European Society of Radiology (ESR) 28 defines clinical audits as a tool designed to improve the quality of patient care, experience, and outcome through formal review of systems, pathways, and outcomes of care against evidence-based standards, and the implementation of change based on the results. Examples of audit areas according to the IIRRT 7 guidelines include the number of IV cannulation attempts per practitioner, the frequency of any extravasation and reactions to contrast media, and any other adverse incidents. This is one area that should be considered during the planning stages of role extension in this area.

In places like Zambia where legal and policy frameworks need to be adjusted to accommodate for role extension, evidence will need to be assembled with respect to four areas; readiness of current practising radiographers to take on additional responsibility, the attitude of medical officers (radiologists and other doctors) towards this approach of improving service delivery, type of additional training required to upskill radiographers, and consultation with patients regarding their acceptability to this initiative. Therefore, research will need to be conducted to better inform decisions concerning the above areas.

### Infection control related to IV cannulation

One study 18 in this review focused on infection control measures when performing IV cannulation by radiographers. Ehrlich and Coakes^27^ defined infection control as measures used to prevent infection through good hand hygiene. However, Suing and Davis 18 most of the radiographers in their study did not consistently wash their hands before and after performing IV cannulation. Alternatively, hand hygiene can be maintained by using an alcohol-based solution for disinfection 29. Whilst elements of infection control are included in the Zambian radiography training programmes, very little emphasis is placed on this aspect because of the perceived non-utilisation of the skills as radiographers are not allowed to perform these tasks in actual clinical practice. Therefore, infection control is one area that should be included in the training programme and local guidelines for radiographers.

### Safety and complications of IV cannulation and administration of contrast media

One study by Munro et al., 19 dealt with the safety and complication of IV cannulation and administration of contrast media. Ehrlich and Coakes^27^ state that the fundamental professional roles of radiographers are focused on providing safe benefits to patients. In the context of this review, the safety issues involve checking that there are no contraindications to contrast media use and ensure that the patient understands that it is to be given and agrees to the procedure except in case of an emergency 7,29,30. In cases where there is a previously reported moderately severe or severe reaction to intravascular contrast, caution should be exercised and the need for the use of contrast should be re-evaluated with respect to an unenhanced study 30. The risk of developing a contrast media reaction after IV iodinated or gadolinium-based contrast is less than 0.7% 31. Any adverse reaction to contrast should be recorded in the patient's records, which should include the name of the contrast media and dose, the details of the reactions, and treatment given 8,27,29. This is one area that should be included in the training programme and local guidelines regarding the prevention, identification, and management of adverse reactions to contrast media.

Out of seven studies included in this review, only one study 20 focused on extravasation in patients undergoing CT examinations. Baheti and others 32 define extravasation as the leakage of intravenously administered contrast media from the normal intravascular compartment into the surrounding soft tissues. The incidence of contrast media extravasation is less than 1% and is usually mild^33^. In the survey conducted in Ireland by Cleary et al., 20 identified patients with fragile veins at high risk of extravasations. The CT angiogram examinations were also identified as a high risk of extravasation. To reduce viscosity and risk of extravasation, the ESUR 29 guideline recommends warming iodine-based contrast media at room temperature (24 °C) or human body temperature (37 °C) before being administered. For non-angiographic examination, the IIRRT guidelines 7, also recommends that any radiographer administering via an automatic injector should monitor the injection and patient during contrast administration via palpation for the initial 15 seconds, followed by visual and/or oral communication with the patient after leaving the CT scanning room.

This review found a lack of agreement on the use of cold or hot packs in the treatment of extravasation. The ESUR 29 guidelines have outlined cold compress as the best treatment approach of extravasation, whilst the American College of Radiologists (ACR) 6 guidelines have stated that there is no clear evidence on which is the better approach. Cold packs may be useful for relieving pain at the injection site, whilst heat packs may help improve absorption of the contrast media 20. Any extravasation should be formally documented, including treatment and advice given to the patient 27,29. The management of extravasation is one area that should be included in the training and local guidelines.

This review found that a radiologist supervising the radiographer undertaking IV cannulation and administering contrast media should be responsible for managing any adverse reactions. This finding agrees with the best practice 7,30. However, if the training of radiographers is enhanced to include prescribing and managing adverse events, the efficiency of the health delivery system can be improved especially in countries like Zambia where a shortage of radiologists exists. Extension of prescription duties to non-medical doctors has already been implemented in fields such as nursing. In several countries, nurse prescriber training and position have been implemented successfully 34. By extension, such an approach can be taken in the radiographer - radiologists relation wherewith adequate training, these roles can be taken up by the radiographers.

This review found no study which focused on post-contrast acute kidney injury (CI-AKI). The previous terminology such as contrast nephrotoxicity (CIN) or radiocontrast nephropathy (RCN) has been replaced with post-contrast acute kidney injury 30,29. The European Society of Urogenital Radiology (ESUR) 29 define post-contrast acute kidney injury as an increase in serum creatinine > 0.3 mg/dl (or > 26.5 µmol/l), or > 1.5 times baseline, within 48–72 hours of intravascular administration of a contrast agent. In other words, is a sudden deterioration in the patient's renal function following the recent injection of contrast media. The increased risk is associated with chronic kidney disease, renal transplant, repeated administration of contrast media, heart failure, and patients aged 75 or older 30. To minimise the risk of CI-AKI, an estimated glomerular filtration rate (eGFR), which is the recommended method to estimate renal function before contrast media administration, should be available 7. Generally, the recommended eGFR in adults should be ≤ 60 µmol/l 15,29. With the foregoing in mind, the training of radiographers should be enhanced to equip them with the necessary competencies to interpret laboratory results to ensure that only patients with acceptable eGFR are exposed to contrast media.

### Medico-legal issues related to IV cannulation and administration of contrast media

The individual responsible for obtaining informed consent from the patient was found to be a concern in our review. However, in a study performed by Koch et al., 22 there was an agreement that a radiographer who is performing the IV cannulation should be responsible for obtaining informed consent from the patient, administer the contrast and remain present during the procedure in case of reactions. This finding matches the available guidelines. 6,7,30 The informed consent must be obtained before the procedure, documented, and kept in the patient's records. Failure to abide by this principle is potentially liable to both legal actions by the patient and action by the regulator 4,7,35. The procedure for obtaining informed consent from the patient is another area that should be included in the IV cannulation training programme and local guidelines for radiographers.

The other area of concern relating to medico-legal issues was who must determine the type and dose of contrast media to administer to patients. A study by Koch et al.,^22^ found that radiologists should be responsible for the decisions regarding the type and dose of contrast media inject to imaging patients. However, where protocols and guidelines regarding dosage are available coupled with essential training in adverse events management, the radiographer doing the administering of contrast would be best placed to assess patients and prescribe the contrast. As the case for nurse prescriber role, this will enhance services especially in resource-constrained environments^34^. The radiologist in this respect plays the role of consultant.

In this review, radiographers were also concerned with extending their role without adequate insurance cover. A study conducted by Zain et al., 11 found a concern amongst radiographers regarding inadequate protection in this extended role. This concern was also found in a study by Munro et al., 19 and the need for malpractice insurance to cover radiographers undertaking this role was suggested. The first cover comes from the employer who is liable for the acts or omissions of its employees if having appropriate education and acting within the scope of practice 25,36. This is called vicarious liability. In the UK, radiographers are also covered through professional indemnity insurance through membership with the Society of Radiographers 25. This is another area that should be discussed with the employers before allowing their radiographers to undertake this extended role.

### Education and training in IV cannulation and administration of contrast media

Education and training were identified in our review as essential before radiographers take up new roles. A study conducted by Koch et al., 21 identified three components of the training programme: theoretical, practical, and an examination. In theory, our review found the need for inclusion of the anatomy and physiology of the vascular system, pharmacology of contrast media and reactions to contrast media, basic and advanced life support; in practicals; a minimum of 10 IV cannulations assisted and 20 unassisted; for the examination; both written and practical. This finding matches the training offered in the Republic of Ireland 37. The 6 months Certificate in IV Cannulation and Administration course offered by University College Dublin (UCD) comprises six elements: online lectures, one-day attendance for taught lectures and practical sessions, submission of departmental IV protocol, a written piece of course work, and a record of clinical practice. This review also found that radiographers who had undertaken training found it effective in their new role 17. This shows the importance of taking formal training before starting to perform this role.

Given the above, the scope of practice and guidelines should state the need for training before a radiographer can undertake this role. For example, the IIRRT 7 guidelines state that “Radiographers undertaking IV cannulation and administration of contrast media must have a certificate of competence”. In the context of this review, certificate of competence refers to evidence of completion of a formal training in a field of expertise. The initial training should be supplemented with continuous professional development (CPD) to maintain and enhance knowledge and skills. This also includes performing this task regularly to maintain an adequate level of competence. For example, in Ireland, a radiographer should undertake a refresher course if not performed IV cannulation for more than 6 months. The syllabus should also be subjected to continuous review to keep up to date with any changes in this area 7.

## Conclusion

The performance of IV cannulation and administration of contrast media to patients undergoing imaging examinations has become the responsibility of radiographers in developed countries. However, role extension is still developing in African countries. This review found that before extending the role of radiographers, the scope of practice should be extended, accredited training programme and local guidelines should be put in place. Thus, professional bodies representing radiographers in countries such as Zambia need to play a leading role in the advocacy for role extension.
